# Sucessful Transition in Rare Endocrine Diseases: Patient Experiences in a French Reference Centre

**DOI:** 10.1111/cen.70016

**Published:** 2025-08-19

**Authors:** Karine Aouchiche, Thierry Brue, Emeline Marquant, Gilbert Simonin, Sarah Castets, Rachel Reynaud, Frederique Albarel

**Affiliations:** ^1^ Department of Paediatrics Assistance Publique‐Hôpitaux de Marseille (APHM), Paediatric Endocrinology Unit, CHU Timone Enfants Marseille France; ^2^ INSERM, MMG, UMR 1251, Institut MarMaRa Aix Marseille Univ Marseille France; ^3^ Department of Endocrinology Centre de Référence des Maladies Rare de l'Hypophyse HYPO, Assistance Publique‐Hôpitaux de Marseille (APHM), La Conception University Hospital Marseille France

**Keywords:** brain tumour, congenital pituitary deficiency, endocrine disorders, experience, growth hormone deficiency, corticotropic deficiency, hypopituitarism, joint consultation, rare disease, transition to adult care

## Abstract

**Objective:**

This study aimed to evaluate the experiences of patients who had a joint endocrinology consultation for transition to adult care at Marseille university hospitals between 2010 and 2020, focusing on patient follow‐up, satisfaction, difficulties, and expectations.

**Methods:**

A healthcare transition questionnaire was designed and administered to patients several years after transition to adult care.

**Results:**

One hundred and fifteen patients with rare endocrine disorders were included, with a mean age of 18.8 years at the transition consultation. Ninety‐six percent (110/115) continued adult care after the first joint consultation, and 75% were still in follow‐up when completing the questionnaire (mean follow‐up, 4.5 years). Of the 81 respondents, 89% were satisfied with the transition, and 64% reported no difficulties. The most common difficulties were psychological, logistical, and medical. Fifty‐three out of 74 respondents (72%) felt the transition occurred at the right time, 17 (24%) thought it was too early, and 4 (5%) felt it was too late. The main concern was the transmission of medical information between doctors. Suggestions for improvement included more joint consultations and personalized transition pathways.

**Conclusion:**

In this group of rare endocrine disease patients, a transition pathway based on a joint pediatric‐adult consultation was associated with high patient satisfaction and long‐term follow‐up rates. Patients' suggestions and reported difficulties highlight issues to be addressed and complementary strategies to develop.

## 1 Introduction

The concept of transition to adult care was first introduced in the 1990s [1] to describe the purposeful and systematic transfer of adolescents and young adults with ongoing physical or medical conditions from child‐centred to adult‐oriented healthcare systems [2]. This process occurs at a challenging period and affects patient follow‐up and health outcomes [3, 4, 5]. Interventions have therefore been developed to improve continuity of care [6] and international guidelines have been proposed [7]. These guidelines focused initially on common diseases [8, 9, 10] before being extended to rare diseases, which require more specific care [11]. Rare diseases are defined in Europe as those with a prevalence below 1 in 2000. Many endocrine disorders fall in this category [12] and require expert care [13]. Patients with rare endocrine pathologies are heterogeneous, including individuals with endocrine tumours or constitutional disorders as well as childhood cancer survivors with endocrine sequelae [14]. A particular focus is required on growth, puberty, and fertility during this period [15]. Good communication between pediatric and adult endocrinologists is crucial for a successful transition to adult care [16]. Although the management of endocrine treatments during this period has been widely studied [17, 18, 19, 20], particularly for growth hormone and puberty induction treatment and progression, patient experiences of this process have rarely been investigated [15, 21].

In our reference centre for rare endocrine diseases, a structured programme has been introduced since 2010, involving joint pediatric‐adult consultations to facilitate patients' transition to adult care. The aim of this study was to evaluate the experiences of young adults with childhood‐onset rare endocrine disorders who went through this process over the past decade.

## 2 Materials and Methods

### 2.1 Patients

This retrospective study included all patients with rare endocrine disease who received a consultation focused on transition to adult care in the presence of a pediatric endocrinologist and an adult endocrinologist in Marseille university hospitals between 1 January 2010 and 31 December 2020. One adult endocrinologist and three pediatricians participated in these consultations. The consultations were scheduled at the request of the pediatrician for patients at least 15 years of age when they and their family were assumed to be ready to transfer to adult care. Consultations were conducted in either the pediatric or the adult endocrinology department, which are in different hospitals but less than 1 km apart. Both departments are part of the national reference centre for rare pituitary diseases (HYPO, also an accredited member of the European EndoERN network) and are accredited competence centres for other rare endocrine diseases as part of the National Rare Diseases Plans initiated by the French Ministry of Health. The consultations lasted between 30 and 45 min. They began with a brief overview of the patient's medical history. Parents were also invited to attend. A follow‐up appointment with the same adult endocrinologist was scheduled at the end of the joint consultation.

### 2.2 Ethics

As a questionnaire survey, this study is considered noninterventional under French law and no specific ethics committee approval was required. The study was registered with the French data protection agency (CNIL, registration number V3U492).

### 2.3 Data Collection

**Figure 1:**
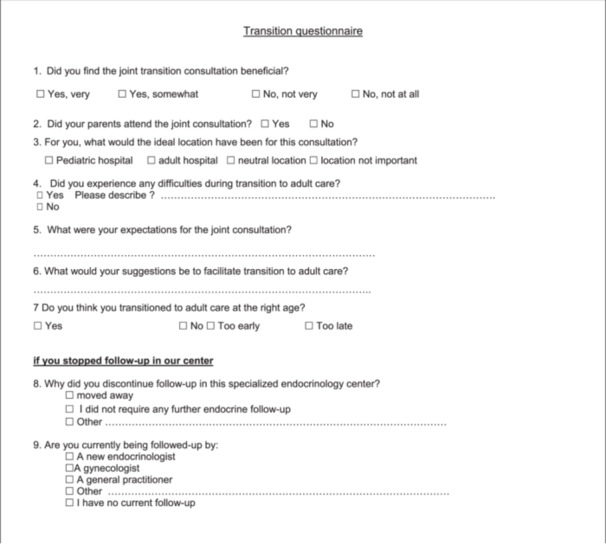
Transition to adult care patient experience questionnaire.

Patient data were collected from electronic medical records, including endocrine disease, weight, height, BMI, treatment, sex, date of joint consultation, and date of last pediatric consultation. A satisfaction questionnaire on the transition consultation was developed based on previous studies of healthcare transition and healthcare experiences (Figure 1). The entire cohort was then contacted between 1 February and 31 March 2021, to complete the questionnaire during a telephone interview. Once informed consent had been obtained, the questionnaire was administered by a single investigator not involved in the patient's care. Depending on the patient's wishes or level of understanding, parents could also participate. Although participants were adults at the time of the interview, they were asked to retrospectively report on their experiences during adolescence and young adulthood, particularly in relation to the transition process.

### 2.4 Statistical Analysis

All statistical analyses were carried out with the software R [22]. Categorical variables were summarized as counts and percentages and continuous variables as mean and/or median values and ranges. Continuous variables were compared between groups using *t* tests. Categorical variables were compared between groups using *χ* ^2^ tests. Differences were considered significant at *p* < 0.05.

## 3 Results

### 3.1 Patient Characteristics at Transition to Adult Care

Patient characteristics at the time of transition to adult care are summarized in Table 1. In total between 2010 and 2020, 115 pediatric endocrinology patients (69 female, 60%) had a joint consultation for transition to adult care. The mean age was 18.8 years (range, 15 to 35 years), and 21 (18%) were older than 20. Disease characteristics are presented in Table 1. Nearly two fifths of patients had a hypothalamic‐pituitary disease (44/115 patients, 39%). Fifteen patients (13%) were not receiving any endocrine treatment at the time of the transition consultation.

**Table 1:** Patient characteristics at the time of the joint transition consultation.

Patients' characteristics	
Sex ratio % (F/M)	1.5 (69/46)
Mean age (range, median)	18.8 (15–35, 18.6)
Mean height in cm (range, median)	
Female (*n* = 69)	159 (141–184, 163)
Male (*n* = 42)	169 (125–188, 164,5)
Mean BMI (kg/m^2^) (range, median)	
Female (*n* = 46)	24.4 (15–37, 23.9)
Male (*n* = 28)	24.5 (15–44, 22.3)
Number of endocrine treatments	
0	15 (13.1%)
1	46 (40.3%)
2	31 (27.2%)
3	12 (10.5%)
4	4 (3.5%)
5	6 (4.2%)

Disease	Number of patients
Brain tumour	21
− Craniopharyngioma	7
− Pituitary adenoma	5
− Germinoma	3
− Astrocytoma	3
− Medulloblastoma	2
− *Unspecified pituitary tumour*	1
Congenital hypopituitarism	14
− Isolated pituitary hormone deficiency	2
− Combined pituitary hormone deficiency	12
Isolated central diabetes insipidus	1
Prader Willi syndrome and hypothalamic obesity	9
Polycystic ovary syndrome^a^	6
Adrenal disease	15
− 21‐hydroxylase deficiency (classic forms)	9
− Other^b^	6
Hypergonadotropic hypogonadism	35
− After treatment for hematological disease^c^	23
− Turner syndrome	7
− Premature ovarian insufficiency	4
− Klinefelter syndrome	1
Difference in sex development^d^	4
Bone disease	3
Thyroid disorder (excluding hematological disease)^e^	7

^a^ With syndromic obesity (*n* = 2), with severe hyperandrogenism (*n* = 3), with anorexia nervosa (*n* = 1).

^b^ Adrenal deficiency (*n* = 3, including after adrenal surgery (*n* = 1)), hypoaldoteronism (*n* = 1), pseudohypoaldosteronism (*n* = 1), 11 hydroxylase deficiency (*n* = 1)

^c^ Allograft transplantation (*n* = 21), thyroid disease (*n* = 12) including thyroid carcinoma (*n* = 3)

^d^ Duplication of DAX‐1 (*n* = 2); androgen insensitivity syndrome (*n* = 2)

^e^ Congenital hypothyroidism (*n* = 4), thyroid carcinoma (*n* = 2), Hashimoto thyroiditis and thyroid nodule (*n* = 1)

### 3.2 Follow‐Up

Of the 115 patients included in the study, one patient with Prader–Willi syndrome died of sudden death during the follow‐up period. Of the remaining 114 patients, 109 (96%) had at least one further adult endocrinology consultation. None returned to the pediatric centre for follow‐up. Among the five patients who only attended the transition consultation, two were being treated for post‐transplant endocrine complications and were also being followed in a reference hematology centre (one of these two patients also moved to another city), two were being treated for polycystic ovarian syndrome in context of syndromic obesity and one had congenital hypothyroidism. One of the patients with polycystic ovarian syndrome and the two patients with post‐transplant endocrine complications were not receiving any treatment.

The mean follow‐up time after the joint transition consultation was 4.5 years (median, 4 years; range, 0.67–10.25 years). Twenty‐three patients (20%) stopped follow‐up, 2 years on average after the transition consultation, at a mean age of 21 years, and after an average of five follow‐up consultations. The 86 remaining patients (75%) were still in follow‐up at the time of the study. Follow‐up status (ongoing vs discontinued) was not associated with age at the time of transition to adult care (*p* = 0.45) or endocrine treatment status (yes/none, *p* = 0.76); however, 10/23 patients who discontinued follow‐up had a hematological disease and this was significantly associated with follow‐up status (*p* = 0.01).

### 3.3 Questionnaire

Of the 114 surviving patients at the time of the study, 31 could not be contacted and 2 declined to respond to the questionnaire. Eighty‐one patients responded to some or all survey questions.

### 3.4 Satisfaction

Seventy of the 79 respondents were either satisfied or very satisfied with the transition process (two patients did not answer this question), giving a satisfaction rate of 87%. Among the nine remaining patients who reported being somewhat dissatisfied, seven stopped follow‐up in 2021 (two immediately after the joint consultation and five after a mean delay of 1.5 years). Six of these nine patients reported experiencing difficulties during transition to adult care.

### 3.5 Transition Pathways

The transition consultation took place in pediatrics for 21 patients (26%) and in the adult endocrinology department for 60 patients (74%). Among the 78 patients who answered the question about the best setting for transfer consultations, just over half (40, 51%) did not consider the location of the consultation important, 9 of whom had had their consultation in pediatrics and 31 in the adult department. Seventeen patients (22%), all of whom had had a joint consultation in the adult endocrinology department, replied that an adult department was best, 18 patients (23%), 10 of whom had had a joint consultation in the adult endocrinology department, replied that a pediatric department was best, and 3 patients (4%), all of whom had had a joint consultation in the pediatric department, replied that a neutral location would have been better. Seventy patients (88%) attended the joint consultation with one or both parents.

Fifty‐three out of 74 respondents (72%) considered that their transition to adult care occurred at the right age, 17 (23%) thought that it had happened too early, and 4 (5%), too late. There was no significant difference in the age of transition to adult care between patients who considered it had happened at the appropriate age (18.7 ± 2.3 years) and those who thought it had happened too early (mean age, 18.8 ± 2.2 years; *p* = 0.7) or with those who considered they had transitioned to adult care too late (mean age, 19.3 ± 1.3 years; *p* = 0.20).

### 3.6 Patient‐Reported Difficulties

The difficulties reported by patients are presented in Table 2. Fifty‐one patients (64%) reported no difficulties at all during the transition to adult care. Patients could classify difficulties they encountered as psychological, logistical, or medical. Psychological difficulties were the most frequently reported, by 18 patients (23%), including apprehension about changing doctors (*n* = 9), difficult relationships with the new doctor, feeling out of step with adult patients, fear of not being sufficiently prepared for the transition, and long‐term monitoring fatigue. Logistical difficulties were reported by 10 patients (13%), mainly hospital orientation and parking. Medically, 5 participants (6%) expressed fear of less personalized follow‐up, fear of miscommunication between pediatric and adult care teams, and difficulties in understanding medical explanations. There was no significant difference in the age of transition to adult care between patients who reported difficulties and those who did not (mean ages, 19.1 ± 2.3 and 18.7 ± 2.3 years respectively; *p* = 0.40).

**Table 2:** Reported difficulties related to transition to adult care.

Psychological	Logistical	Medical
Anxiety about changing doctors (*n* = 9) Difficult relationship with new doctor (*n* = 4) Feeling out of step with adult patients (*n* = 2) Insufficient preparation (*n* = 2) Tired of hospital follow‐up (*n* = 1)	Directions, parking (*n* = 9) Doctors in different hospitals (*n* = 1)	Anxiety about less personalized follow‐up in adult medicine (*n* = 2) Fear of receiving incorrect medical information (*n* = 2) Poor understanding of medical explanations (*n* = 1)

### 3.7 Reasons for Stopping Follow‐Up

Nineteen patients left follow‐up between the transition consultation and answering the questionnaire (mean delay, 4.5 years). Eight of these patients reported no further need for endocrinological follow‐up as the reason for stopping (7/8 of these patients had not been receiving any endocrine treatment at transition to adult care) and 6 reported moving to a different city. Among the five remaining patients, two reported that they wished to continue follow‐up closer to home, two reported experiencing difficulties with their new doctor, and one reported difficulty making appointments. Of the 19 patients who left follow‐up, five had no follow‐up after the joint consultation, nine were being followed‐up by a different endocrinologist, two by a gynecologist, one by a hematologist and two by their general practitioner.

### 3.8 Expectations

Patients' expectations are summarized in Table 3. Nearly half of respondents (37/79, 47%) stated that they had no specific expectations about the transitional consultation, and 72% (57/79) did not suggest any improvements, stating that the consultation structure was good in 54% of cases (43/79). Patients' suggestions are listed in Table 4. The main suggested improvements were multiple joint consultations, personalized transition pathways, remaining in the same hospital and having medical discussion about fertility and healthy eating.

**Table 3:** Patient expectations for the transition consultation.

Expectations
Transmission of medical information to the new doctor (*n* = 23) Meeting the new doctor (*n* = 16) Explanations about their disease (*n* = 7) Explain ongoing treatments (*n* = 4) Treatment changes (*n* = 4) Reassurance about the period of transition (*n* = 2) No specific expectations (*n* = 37)

**Table 4:** Patient suggestions to improve transition to adult care.

Suggestions
Remaining in the same hospital (*n* = 4) Multiple joint consultations (*n* = 4) More personalized transition pathway (*n* = 4) Discussing fertility and healthy eating (*n* = 4) Improved scheduling of medical appointments (*n* = 3) More gradual transition to adult care (*n* = 3) Help feeling comfortable with the doctor (*n* = 1) More understandable medical explanations (*n* = 1)

## 4 Discussion

In pediatric chronic disease management, effective transition to adult care is crucial to limit patient drop‐out and the associated negative consequences. Gabriel and colleagues point out that the success of this process depends on factors such as population health, patient experiences of care and care utilization and costs [4]. In endocrinology, recommendations for transition focus primarily on treatment [18] while the experiences of patients with rare endocrine diseases transitioning to adult care have received little attention [19].

In this study, we evaluated the experiences of 115 patients with rare endocrine diseases who transitioned to adult care in our hospitals over 10 years, from questionnaire responses collected with a median of 4 years after their joint pediatric‐adult transition consultation. The main results of this study are that 89% of patients found the joint consultation beneficial and 64% reported no difficulties in their transition to adult care. These results are consistent with previously reported satisfaction rates of 80% and 84% with transition processes [3, 23]. In our study, the positive questionnaire responses are supported by the fact that 95% of patients attended at least one further follow‐up consultation with the adult endocrinologist and by the fact that 75% of patients were still being followed up at the time of the study, 4.5 years on average after the transition consultation. This is nearly as high as observed in a large French cohort of rare endocrine disease patients, where 88% were still in follow‐up 21 months on average after transitioning to adult care [21]. In this cohort, a number of measures were implemented to avoid drop‐out, including patient recall procedures managed by a transition coordinator. Our results in a real‐world setting with no additional interventions are therefore particularly encouraging. In our study, the most common reason for stopping endocrinological follow‐up was concurrent follow‐up with a different physician. For instance, patients being followed up by a hematologist after leukemia treatment did not consider their endocrinologist their main doctor and these patients were the most likely to stop endocrine follow‐up. This result suggests that the importance of long‐term endocrinological follow‐up should be established with patients from the onset of the transition period, and that multidisciplinary care pathways should be emphasized, for example via joint multidisciplinary consultations.

Transition to adult care is a gradual process and a moment of vulnerability in the patient's journey; therefore it cannot be tackled by a simple transfer consultation [2]. According to a 2016 consensus report on transition to adult care in endocrine pathologies, this should begin around the ages of 11–14 years, and the timing should be adjusted to the pathology [13]. The joint consultation involving a pediatrician and an adult specialist appears to be a pivotal step in this process [24]. In our study, the timing of the transfer consultation was based on an assessment patients' maturity by their pediatrician, family members and patients themselves. Before the transfer consultation, the pediatrician organized a separate appointment with their patient alone to assess their degree of autonomy in managing their condition. If necessary, a patient education programme was offered ahead of the joint consultation. Nearly three quarters of patients (72%) thought that the timing of their joint transition consultation was appropriate. Most of the remaining patients (23%) thought that it had been organized too soon, and only 5% too late. This outcome was not associated with patient age at transition in contrast with studies that only focused on the age of transition [25]. Our findings emphasize the importance of individualized pathways. This could be improved by integrating personalized care questionnaires [26] into the transition process.

More than half of the respondents in our study did not consider the location of the transition consultation to be important. This result differs from those of two previous studies that underline the significance of a suitable transition environment for adolescents and young adults, proposing “transition clinics” as the ideal setting [27, 28]. A new facility has recently been created between the pediatric and adult departments in our hospitals to host consultations and patient education programmes for adolescents and young adults with chronic illnesses. An evaluation of patient experiences in this context would be of interest and could complete our study.

This study took place in an accredited reference centre for rare endocrine diseases, one of whose main objectives is the promotion of interactions between pediatric and adult specialists to improve continuity of care during transition to adult care [29]. In our centre, these specialists work together on a daily basis, which is particularly valuable during the transition process. These settings may therefore have contributed to the positive patient experiences and follow‐up outcomes reported.

Regarding patient expectations, difficulties and areas for improvement, the main reported expectation of the transition process was that the transfer of medical information to adult doctors should be as clear and complete as possible. The joint consultations began with a review of the patient's medical history in their presence, accompanied by a written summary, allowing patients to add details or make corrections as needed. At the end of consultation, patients were provided with a summary, as currently recommended [30].

A limitation of our study is that it does not assess the impact of family support during the transition period. Allen and colleagues and Badour and colleagues have highlighted the importance of involving parents in this process [31, 32]. Parents may also find their child's transition to adult care challenging [33]. It would therefore be interesting to extend the present study with a cross‐evaluation of parental involvement, difficulties encountered in this regard and parent experiences during the transition process.

A meta‐analysis of 122 medical organizations involved in healthcare transition since 1999 and a more recent study in a French endocrine reference centre identified human and financial resources as the main obstacles to the implementation of transition programmes [21, 34, 35]. Similarly, within our centre, we face challenges in advancing the transition process due to human and financial resource limitations. Another limitation of our study is that our questionnaire did not include questions about the human resources (psychologists, doctors, transition coordinators) necessary to optimize the transition process.

## 5 Conclusion

This study on the transition to adult care is the first to specifically focus on patient experiences in rare endocrine diseases. The positive assessments of the transition process by 89% of patients and the low dropout rate after transition suggest that the care organization in French reference and competence centres for rare diseases plays a crucial role in facilitating healthcare coordination, patient integration into adult care, and minimizing follow‐up cessation. The results of our study highlight key expectations for improving the transition process like multiple joint consultations, personalized transition pathways, remaining in the same hospital, and having medical discussions about fertility and healthy eating. Our findings suggest that further improvements in follow‐up rates could be achieved by expanding these personalized care pathways and developing patient education programmes. Our centre is thus developing a specific therapeutic education programme for the transition period. Furthermore, the development of joint guidelines by European pediatric and adult endocrinology societies highlights the growing importance of this issue, emphasizing the significance of our study in improving the transition process.

## Ethics Statement

All participants provided consent for participating. The study was registered with the French data protection agency (CNIL, registration number V3U492).

## Conflict of Intersest

The authors declare no conflicts of interest.

## Data Availability

All data generated or analyzed during this study are included in this article. Further inquiries can be directed to the corresponding author.
